# Applicability of the Compensatory Encoding Model in Foreign Language Reading: An Investigation with Chinese College English Language Learners

**DOI:** 10.3389/fpsyg.2017.00681

**Published:** 2017-05-04

**Authors:** Feifei Han

**Affiliations:** Centre for Research on Learning and Innovation, Sydney School of Education and Social Work, The University of Sydney, Sydney NSW, Australia

**Keywords:** Compensatory Encoding Model, word recognition, working memory, reading comprehension, foreign language reading, Chinese college English language learners

## Abstract

While some first language (L1) reading models suggest that inefficient word recognition and small working memory tend to inhibit higher-level comprehension processes; the Compensatory Encoding Model maintains that slow word recognition and small working memory do not normally hinder reading comprehension, as readers are able to operate metacognitive strategies to compensate for inefficient word recognition and working memory limitation as long as readers process a reading task without time constraint. Although empirical evidence is accumulated for support of the Compensatory Encoding Model in L1 reading, there is lack of research for testing of the Compensatory Encoding Model in foreign language (FL) reading. This research empirically tested the Compensatory Encoding Model in English reading among Chinese college English language learners (ELLs). Two studies were conducted. Study one focused on testing whether reading condition varying time affects the relationship between word recognition, working memory, and reading comprehension. Students were tested on a computerized English word recognition test, a computerized Operation Span task, and reading comprehension in time constraint and non-time constraint reading. The correlation and regression analyses showed that the strength of association was much stronger between word recognition, working memory, and reading comprehension in time constraint than that in non-time constraint reading condition. Study two examined whether FL readers were able to operate metacognitive reading strategies as a compensatory way of reading comprehension for inefficient word recognition and working memory limitation in non-time constraint reading. The participants were tested on the same computerized English word recognition test and Operation Span test. They were required to think aloud while reading and to complete the comprehension questions. The think-aloud protocols were coded for concurrent use of reading strategies, classified into language-oriented strategies, content-oriented strategies, re-reading, pausing, and meta-comment. The correlation analyses showed that while word recognition and working memory were only significantly related to frequency of language-oriented strategies, re-reading, and pausing, but not with reading comprehension. Jointly viewed, the results of the two studies, complimenting each other, supported the applicability of the Compensatory Encoding Model in FL reading with Chinese college ELLs.

## Introduction

Most of us read everyday, from academic texts to technical reports, from literature to popular magazines, and from newspapers to brochures. The seemingly common practice of reading in fact is a highly complex cognitive activity (Koda, 2007; Yamashita, 2013), even when reading in one’s first language (L1), let alone reading in a foreign language (FL). One of the major obstacles faced by FL readers is slow word recognition, which requires much conscious deliberation (Segalowitz, 2000, 2003; Fukkink et al., 2005; Segalowitz and Hulstijn, 2005). Inefficient word recognition takes much cognitive resources, such as working memory, which is essential for reading comprehension to occur (Juffs and Harrington, 2011). Some theoretical models of reading, such as the Verbal Efficiency Model highlights the importance of word recognition efficiency and working memory, suggesting that inefficiency in word recognition and small working memory tend to inhibit higher-level comprehension processes (e.g., Perfetti, 1985, 2007). On the other hand, in the Compensatory Encoding Model, the role of strategic processing is emphasized and the model postulates that as long as readers have sufficient time to carry out a reading task, slow word recognition and limited working memory do not normally hinder reading comprehension, because readers are able to apply some kinds of higher-order metacognitive strategies to remedy processing efficiency (slow word recognition) and resource limitation (small working memory), and that is to say those metacognitive strategies have compensatory characteristics (Walczyk, 2000; Walczyk et al., 2001, 2007).

The Compensatory Encoding Model of reading was proposed by Walczyk and his colleagues to explicate “the interplay between automatic and control processes” (Walczyk, 2000, p. 35) in L1 reading beyond the initial stages of learning to read (Walczyk, 1993, 1995; Walczyk and Taylor, 1996; Walczyk et al., 2001, 2007). The construction of the model is based on a number of L1 reading theories (Walczyk, 2000), including Automaticity Theory (LaBerge and Samuels, 1974), the Verbal Efficiency Model (Perfetti, 1985, 1988), Metacognitive Theory (Baker and Brown, 1984), Constructively Responsive Theory (Pressley and Afflerbach, 1995), and Rauding Theory (Carver, 1997). According to the model, in fluent reading, processes such as identifying words and accessing to meanings tend to be carried out automatically, which make few demands on working memory. As a result, working memory can be freed up for higher-level comprehension processes, which are operated in slow, error prone, unstable, and serial manner (Walczyk, 2000; Shiotsu, 2009). In situations where readers have processing limitation (i.e., inefficient word recognition) and have resource limitation (i.e., small working memory), the model highlights the importance of compensatory mechanisms, which are metacognitive strategic processing (Walczyk, 1995). The model postulates that the condition for compensatory mechanisms to operate successfully during reading relies heavily on reading time: when there is severe time constraint, such as in a testing situation, it is less likely for readers to operate compensatory mechanisms freely. The application of mechanism as successful compensation for inefficient word recognition and small working memory tends to occur when reading without much time constraint (Walczyk, 1993, 1995, 2000; Walczyk and Taylor, 1996; Walczyk et al., 2001, 2007).

The Compensatory Encoding Model entails two important predictions. The first prediction is that reading conditions varying time influence the relationship between word recognition efficiency, working memory, and reading comprehension. When reading without time constraint, inefficient word recognition and small working memory do not normally affect reading comprehension “because compensatory mechanisms operate routinely during performance” (Walczyk, 1993, p. 127). That is to say there will be only a weak or no relationship between word recognition efficiency, working memory, and comprehension. When reading is under time constraint, compensatory mechanisms are less likely to operate freely; hence, inefficient word recognition and small working memory tend to be adversely affects reading comprehension (Walczyk, 1993, 1995, 2000; Walczyk and Taylor, 1996; Walczyk et al., 2001, 2007).

The second prediction is that when reading occurs without time constraint, it is the use of compensatory strategies which are predictive of reading comprehension. This means that readers with slower word recognition and smaller working memory tend to use more metacognitive mechanism in reading without time constraint, and as a result, their reading comprehension tends not to be affected by word recognition inefficiency and limited working memory (Walczyk and Taylor, 1996).

To empirically test the Compensatory Encoding Model, Walczky and his associates conducted a series of studies with both young and mature native English speakers. In one of the earlier studies, Walczyk (1995) compared contributions made by word recognition and working memory to reading comprehension with and without time constraint among university students. The results showed that without time constraint, none of the measures of word recognition and working memory related to reading comprehension. However, when reading under time constraint, word recognition efficiency and working memory were significantly associated with comprehension. This study provided evidence for the first prediction in the Compensatory Encoding Model among adult L1 readers.

To test the actual use of compensatory strategies in reading (the second prediction), Walczyk and his colleagues conducted a few studies with children and adults. Some of these studies provided full support (e.g., Walczyk et al., 2004), whereas some studies only provided partial support for the Compensatory Encoding Model, especially among younger children (e.g., Walczyk et al., 2007). Among primary school students, Walczyk et al. (2004) recorded the participants’ reading aloud of one narrative and one expository text by giving children sufficient time to read. They found that measures of word recognition efficiency and working memory were significantly and negatively related to frequency of using pausing and re-reading – two kinds of compensatory strategies, in reading both types of texts. Walczyk and his associates also found some support for the Compensatory Encoding Model among adult readers (e.g., Walczyk and Taylor, 1996; Walczyk et al., 2001). For instance, Walczyk and Taylor (1996) asked university students to read texts on a computer screen without time constraint and recorded students’ re-reading behaviors using a computer program. Their re-reading was found to be significantly correlated with the speed measures of word recognition and working memory, suggesting that readers with inefficient word recognition and small working memory tended to re-read more frequently.

Using the think-aloud method, which allowed readers to process reading task at hand with ample time, Walczyk et al. (2001) found that slower word recognition was associated with more frequent pausing, looking back, and re-reading behaviors, and slower speed measure of working memory was associated with more re-reading behaviors. In addition, neither of the speed measure of word recognition nor working memory was related to reading comprehension, implying readers’ compensatory use of pausing, looking back, and re-reading strategies in reading.

A further examination of the Compensatory Encoding Model investigated developmental pattern in the relationship between word recognition, working memory in relation to use of compensatory strategy among third, fifth, and seventh graders (Walczyk et al., 2007). The researchers manipulated the reading conditions by placing time restrictions to create either time restricted reading or non-time restricted reading and students were randomly assigned to one of the reading conditions. In the non-time restricted reading, students were asked to read-aloud to enable coding of possible compensatory strategies, such as pausing, looking back, and jumping over. The results demonstrated that the relational pattern between word recognition, working memory, and use of compensatory reading strategies exhibited a consistent pattern across the three grades: both the accuracy measure of word recognition and working memory were negatively correlated with jumping over for third and seventh graders, and with looking back for fifth graders, indicating that slower word recognition and smaller working memory appeared to be associated with more frequent application of compensatory mechanism in reading.

However, the relationship between word recognition, working memory, and reading comprehension in the non-time restricted reading condition displayed different patterns for different grades. While word recognition and working memory was found to adversely affect reading comprehension for the third and fifth graders, they did not affect reading comprehension for the seventh graders. The lack of relation for seventh graders seemed to indicate that strategy use as successful compensation was only realized among older and experienced readers, who were more metacognitively and strategically oriented than younger and less experienced readers. The developmental pattern of the results are in line with the creation of the model, which targets more experienced readers, who are able to orchestrate metacognitive reading strategies strategically.

In summary, there was ample empirical evidence which supported the two predictions of the Compensatory Encoding Model in L1 reading: (1) reading conditions varying time affect the relation between word recognition, working memory, and comprehension; (2) in non-time constraint reading, experienced L1 readers displayed compensatory nature of reading strategy use for word recognition inefficiency and working memory limitation.

In FL reading, testing of the Compensatory Encoding Model is lacking. To the best of our knowledge, the only study which has directly investigated the Compensatory Encoding Model in FL reading is conducted by Stevenson (2005) with 22 Dutch adolescent English language learners (ELLs). Stevenson (2005) measured word recognition speed using a computerized lexical decision task, and adopted a think-aloud method to measure concurrent reading strategy use. Reading while thinking-aloud gave readers sufficient time to process a text, which simulated non-time constraint reading condition for the use of reading strategies as a remedy of word recognition inefficiency and limited working memory capacity in the Compensatory Encoding Model. The coding of reading strategy use were broken down into three dimensions, namely orientation of processing (language and content), type of processing (metacognitive, cognitive, and cognitive-iterative), and domain of processing (above clause, clause, and below clause). The results showed that word recognition speed correlated with language-oriented strategies (i.e., strategies directed toward linguistic information), cognitive strategies (i.e., strategies involving direct mental processing of a text), and clause and above-clause level strategies (i.e., strategies which help understand whole or a few successive clauses), but not with reading comprehension. These results provided empirical evidence that in the non-time constraint reading, similar to L1 readers, FL readers are able to deploy compensatory use of reading strategies for slow word recognition so that word recognition did not adversely affect reading comprehension and frequency of strategy use which are characterized as compensatory nature enabled them to achieve reasonable comprehension.

A number of issues in Stevenson’s (2005) study warrant further testing of the Compensatory Encoding Model in FL reading. First of all, while Stevenson’s study focused on investigating the second prediction in the Compensatory Encoding Model, it did not directly examine the first prediction, that is reading conditions varying time affects the relation between word recognition, working memory, and comprehension in FL reading. Secondly, working memory was not examined in the study, so it was not able to draw any direct conclusion regarding the relationship between FL readers’ resource limitations, strategy use, and reading comprehension. Thirdly, the word recognition measure in Stevenson’s study was not appropriate for FL readers, as it only involved decoding but meaning access. For FL readers, decoding may activate a connection to meaning or only leads to a weak connection to meaning (Shaw and McMillion, 2008; Grabe, 2009). Thus, it is more appropriate to use a task which requires meaning access through identification of word forms to measure word recognition efficiency among FL readers. In addition, Dutch ELLs speak a L1 which is typologically close to English. The similarity between the two languages may pose little difficulty in English word recognition. Considering ample evidence of qualitatively different cognitive processes for word recognition by alphabetic and non-alphabetic learners (Koda, 1994, 1996, 2005, 2007), it is necessary to examine whether FL readers whose L1 is a non-alphabetic language, such as Chinese, are able to deploy compensatory use of reading strategy for word recognition inefficiency and working memory limitation as those native English speakers and those ELLs whose L1 is also an alphabetic language.

The present research aims to test the applicability of the Compensatory Encoding Model in FL reading with Chinese ELLs. Two studies were conducted, each focusing on testing one of the important predictions in the Model. Study one aimed to test whether reading condition varying time affects the relationship between word recognition efficiency, working memory, and reading comprehension. The research questions for study one are: (1) To what extent does word recognition efficiency and working memory relate to reading comprehension in the time constraint and non-time constraint FL reading? (2) To what extent does word recognition efficiency and working memory contribute to reading comprehension in the time constraint and non-time constraint FL reading? According to the Compensatory Encoding Model, we hypothesized that in FL reading, when the reading condition is strictly time limited, both word recognition efficiency and working memory tend relate to and contribute to reading comprehension. When reading condition allows readers to have ample time to complete a reading task, word recognition efficiency and working memory tend not to associate with and contribute to reading comprehension.

Study two aimed to examine whether Chinese ELLs are able to operate metacognitive reading strategies as a compensatory way of reading comprehension for inefficient word recognition and working memory limitation in non-time constraint reading. The research question for study two is: What is the interrelationship between word recognition efficiency, working memory, use of reading strategies, and reading comprehension in non-time constraint FL reading?

## Study One

### Material and Methods

#### Participants

The participants in study one were 402 second year undergraduates (138 males and 266 females) recruited from a national university in China. We targeted second year students because first year students had just entered the university, and students beyond second year are not required to be enrolled in the compulsory college English learning (Hu, 2005). The recruitment focused on non-English major undergraduates, as English majors are not representative of the majority of Chinese ELLs, due to their presumably better proficiency and greater interest in English learning. The participants came from 12 English classes, majored in eight disciplines (i.e., Economics and Business, Humanities and Social Sciences, Information Technology and Computer Science, Material Engineering, Mechanical Engineering, Science, Printing and Packaging Technology, and Water Resources and Hydraulic Power). Their age ranged between 18 and 22 with a Mean (*M*) of 20.22 and a Standard Deviation (*SD*) of 0.93. On average, the participants received 7.5 years of English instruction.

#### Materials

##### Word recognition test and scoring

To measure word recognition efficiency, we used a computerized test, which required learners to decide as quickly as possible whether a pair of words had a similar meaning (synonyms) or had opposite meanings (antonyms). The format of the test was adapted from Haynes and Carr’s (1990) paper-and-pencil test and it was essential for the participants to access the meaning of words using this test to measure word recognition. The testing items were 60 word pairs of four different parts of speech (i.e., noun: 14, verb: 20, adjective: 20, and adverbs: 6). To avoid testing vocabulary knowledge of the ELLs, all the testing items were from the most frequent 2,000 words band in the British National Corpus word list (Nation, 2004). The lexical relationship between the words (synonyms or antonyms) was checked using an online thesaurus^1^.

The test which was delivered through Lenovo computers with 17 inch screen with Windows XP system using DMDX software (version 3.3.1.1) (Forster and Forster, 2003) was held in a quiet computer laboratory. Students were required to make a judgment as quickly as possible by pressing two keys marked with Synonym or Antonym. The order of the testing items was randomized using the random function of the software. The test instructions were given in Chinese, and the test started with six practice pairs (see Appendix 1 for sample items).

As the word recognition test aimed to measure students’ efficiency of recognizing English words rather than to test their English vocabulary knowledge, only values of reaction time rather than correctness of judgment were used for data analysis. As in most reaction time analyses, the upper and lower thresholds were set as 3 *SD*s above and below the *M* reaction time of each item (e.g., Muljani et al., 1998; Koda, 2000; van Gelderen et al., 2004). The values of reaction time falling outside the thresholds were located and transformed into missing values. The missing values (accounting for 0.94%) were estimated by using the Expectation Maximization algorithm. After the estimation, the Cronbach’s alpha was calculated and its value was 0.94, indicating good reliability.

##### Working memory test and scoring

We used a modified computerized Operation Span Task, which was developed by Unsworth et al. (2005). The reasons for choosing the OST over the popular Reading Span Task (RST) were: (1) the OST does not involve testing the participants’ reading comprehension ability whereas the RST requires readers to process sentences for comprehension (Kintsch, 1998; Seigneuric et al., 2000; Koda, 2005; Alptekin and Erçetin, 2010, 2011; Rai et al., 2011); (2) the OST tends not to be influenced by language proficiency (Service et al., 2002), whereas the RST if tested in a FL, is affected by levels of proficiency in that FL; and (3) the OST, which is a well-established measure of working memory in the field of psychology with confirmed validity and reliability (Conway et al., 2005; Unsworth et al., 2005), examines both storing and processing functions of working memory simultaneously (Baddeley, 2006, 2007; Juffs and Harrington, 2011).

The OST asked participants to memorize isolated English words displayed on the computer screen for half a second at the same time to judge the correctness of a simple mathematical equation involving addition, subtraction, multiplication, and/or division (e.g., (10 × 5) - 20 = 30) by pressing a key marked with Correct or Wrong. The test was also delivered using the DMDX software (version 3.3.1.1) (Forster and Forster, 2003) via the same computers as used for the word recognition test. There were 40 items (i.e., one item consists of a word for recall and a mathematical equation for judgment) divided into 10 sets ranging from 2 to 6 items in each set. After each set, when the computer displayed “Recall and write down the words within the set (in Chinese),” the participants were asked to write the words on an answer sheet. Upon completion of writing, they needed to press the Space key to proceed to the next set. The words for recall (20 nouns and 20 verbs) were from the most frequent 300 words in British National Corpus word list (Nation, 2004), and all the words were only one syllable ranging from 4 to 6 letters. The task instructions were given in Chinese, and the test started with 14 practice items divided into 4 sets (see Appendix 2 for sample items).

To score the working memory test, we used composite *Z*-scores, which were formed by averaging *Z*-scores of: (1) the number of correctly recalled words, (2) the number of correct judgment, and (3) the reaction time of the judgment (Waters and Caplan, 1996). For the reaction time of the judgment, we trimmed the data using the thresholds of 3 *SD*s above and below the *M* reaction time of each item. The outlying values of reaction time were marked as missing values (accounting for about 1%), and were estimated by the Expectation Maximization algorithm. We multiplied the reaction time by -1 in order for a higher value to represent better performance and then transformed them into *Z*-scores (Leeser, 2007). The reliability, which was calculated using the composite *Z*-scores, had a value of 0.85, indicating that the working memory test was reliable.

##### Reading comprehension tests and scoring

Two parallel reading comprehension tests in the two reading conditions (i.e., time-constraint vs. non-time constraint) were customarily compiled using four expository texts. As research has shown that text type influences reading strategy use (Alderson, 2000; Horiba, 2000; Grabe, 2009; Alptekin and Erçetin, 2011), a decision was made to use a single text type to avoid text type being a confounding factor. Expositions were chosen because they were most familiar to the participants according to the participants’ English teachers. The four texts were adapted from *College Reading Workshop* (Malarcher, 2005), a reading practice book targeting upper-intermediate learners of English. We chose texts based on the following criteria by consulting the participants’ English teachers: (1) the linguistic difficulty was not overly challenging in terms of lexical and morphosyntactic complexity; (2) understanding the texts did not require specialized background knowledge; and (3) the texts were interesting for the participants to read.

To ensure that the two tests had a similar level of readability, the following efforts were made. (1) The topics of the texts in the two reading conditions were matched: both tests had one text related to human body [Text 1 (T1): *Fat to Store or Fat to Burn?*, Text 3 (T3): *Ideas about Beauty*]; and the other related to technology [Text 2 (T2): *Commerce through the Internet*, Text 4 (T4): *A Second Look at Virtual Advertisements*]. (2) Each text had six paragraphs and similar word counts (T1 to T4: 588, 594, 588, and 596 words). (3) The four texts were comparable in terms of T-units and average number of words per T-unit (T1: 29 T-units, 20.24 words/T-unit; T2: 29 T-units, 20.38 words/T-unit; T3: 29 T-units, 20.34 words/T-unit; and T4: 29 T-units, 20.00 words/T-unit). (4) The four texts had comparable Flesch-Kincaid Grade level (T1 to T4: 9.40, 10.90, 9.80, and 10.90). The Flesch–Kincard Grade level is a commonly used readability index for native English speakers rather than ELLs, our purpose of using it was only to check whether the four texts had similar levels of readability.

Reading comprehension questions were multiple choice, which is the most commonly used approach for assessing reading comprehension (Kendall et al., 2001; Brantmeier, 2005; Alptekin, 2006; Phakiti, 2007; Alptekin and Erçetin, 2011). Using multiple choice has a number of advantages over other methods (Brantmeier, 2005; Phakiti, 2007): (1) it is objective and does not require training for scoring; (2) it is convenient to administer to large numbers of students; (3) it does not make heavy demands on readers’ memory, writing ability, and synthesizing skills, compared with recall and cloze tasks (Jenkins et al., 2004; Johnson et al., 2005; Alptekin, 2006; Alptekin and Erçetin, 2011). Although multiple choice question has been criticized for guessing (Alderson, 2000), we informed students that the tests were not part their assessment for the course so that they should avoid guessing.

For each text, 10 multiple choice questions with four possible choices were constructed. Five of them were literal comprehension questions requiring specific information directly stated in the text; and five were inferential questions measuring global text comprehension, such as theme and aims of the text. A correct answer for one question received one point, and the maximum achievable score for each test was 20.

#### Data **C**ollection **P**rocedure

The participants were allowed to spend 40 min to complete the reading comprehension test in the time constraint reading condition. The time was decided by allowing 60% of the average normal reading time of 100 students in a pilot study following Walczyk (1995) and Walczyk and Taylor (1996)’s suggestion. The students in the pilot study had similar level of reading proficiency as the participants. The two reading comprehension tests were conducted on 1 day in the students’ classrooms. Students completed the word recognition and working memory tests on a separate day in a computer laboratory. The 402 participants were grouped into 20 groups with approximately 20 students in each group. Students in each group started the tests at the same time but the completion time varied among them. On average, they spent 30 min to complete the two tests.

#### Data Analysis

To answer research question 1 – the relationship between word recognition, working memory, and reading comprehension in the time constraint and non-time constraint reading conditions, correlation analyses were applied separately for the two reading conditions. To answer research question 2 – the contribution of word recognition and working memory to reading comprehension in the time constraint and non-time constraint reading conditions, regression analyses were performed.

### Results

#### Results of Research Question 1

**Table 1** presents the descriptive statistics of all the tests in study one. The descriptive statistics of the word recognition test are reaction time in milliseconds, descriptive statistics of the working memory test are composite *Z*-scores comprised of accuracy of judgment, the reaction time of judgment, and the number of correctly recalled words.

**Table 1:** Descriptive statistics of all the tests in study one.

Variables	*M*	*SD*	Minimum	Maximum
Word recognition (*in milliseconds*)	1981.06	463.59	1026.95	3984.58
Working memory	0.00	0.19	-0.78	0.40
Comprehension in time constraint condition	12.99	2.88	2.00	19.00
Comprehension in non-time constraint condition	15.14	2.42	2.00	20.00

**Table 1** shows that the *M* scores of the non-time constraint reading comprehension were higher than that in the time constraint reading. A one-way repeated ANOVA revealed that the difference was significant, *F*(1,401) = 127.48, *p* < 0.01, indicating that when FL readers read without time constraint, they achieved better comprehension, even though the texts in the two reading conditions were matched for the level of readability and topics.

The results of the correlation analyses are displayed in **Table 2**, which shows a small and negative correlation between word recognition and reading comprehension in the time constraint condition (*r* = -0.22, *p* < 0.01), indicating that the slower one’s word recognition (hence longer reaction time) was, the poorer one’s comprehension was in the time constraint reading. However, we found that the correlation between word recognition and reading comprehension in the non-time constraint condition was not significant (*r* = -0.09, *p* = 0.07), suggesting that when the participants were allowed to read with sufficient time, the speed of recognizing English words did not affect their reading comprehension.

**Table 2:** Results of correlation analyses.

Variables	Working memory	Comprehension in non-time constraint reading	Comprehension in time constraint reading
Word recognition	-0.28^∗∗^	-0.09	-0.22^∗∗^
Working memory	-	0.11^∗^	0.20^∗∗^

*^∗∗^*p* < 0.01, ^∗^*p* < 0.05, (two-tailed)*.

**Table 2** further shows that working memory was significantly and positively correlated with reading comprehension in both time constraint (*r* = 0.20, *p* < 0.01) and non-time constraint reading (*r* = 0.11, *p* < 0.05). To compare the strength of the correlations between working memory and reading comprehension in the two reading conditions, we used Steiger’s (1980) *Z*-test, and the results indicated that the strength of the correlation in the time constraint reading was significantly stronger than that in the non-time constraint reading, *Z* = 1.71, *p* < 0.05. The results indicated that readers with larger working memory capacity tended to be more strongly related to better reading comprehension in time constraint reading than in non-time constraint reading.

#### Results of Research Question 2

As the word recognition did not correlate with reading comprehension in the non-time constraint condition, the reading comprehension was regressed on working memory only and the results are presented in **Table 3**. **Table 3** revealed that even though working memory significantly contributed to the regression model: *F*(1,400) = 2.26, *p* < 0.05, *f^2^* = 0.01, it accounted for only 1% of variance in reading comprehension in non-time constraint reading with negligible effect size, suggesting the rather weak predictive power of working memory capacity to comprehension in the non-time constraint reading.

**Table 3:** Results of simple regression analysis.

Variables	B	β	*t*	*p*	*f^2^*
Constant	0.76	-	125.14	0.00	-
Working memory	0.07	0.11^∗^	2.26	0.02	0.01

For the time constraint condition, the reading comprehension was regressed on word recognition and working memory. Before performing the multiple regression analysis, we conducted a series of tests to examine the essential assumptions for reliable results. First, the correlation between the two predictors (word recognition and working memory) was 0.28, and the values of Tolerance for word recognition = 0.92, and working memory = 0.92; these ensured no multicollinearity. Second, the analysis of standard residuals on the data identified four cases as outliers (falling out of ±3 *SD*s), which were removed (less than 1% of the data). Third, the value of the Durbin–Watson was 1.86, suggesting that there was no auto-correlation in our data. After confirmed that our data met these assumptions, we proceeded with the multiple analysis. The results were presented in **Table 4**, which shows that both word recognition (β = -0.18, *R^2^* = 0.05, *p* < 0.01, *f^2^* = 0.05) and working memory (β = 0.15, *R^2^* = 0.02, *p* < 0.01, *f^2^* = 0.02) significantly contributed to reading comprehension in the time constraint reading, explaining 5 and 2% of variance respectively.

**Table 4:** Results of multiple regression analysis.

Variables	B	β	*t*	*p*	*f^2^*
Constant	0.67	-	89.05	0.00	-
Word recognition	-0.10	-0.18^∗∗^	-4.53	0.00	0.05
Working memory	0.11	0.15^∗∗^	2.92	0.00	0.02

**R^*2*^* = 0.01, ^∗^*p* < 0.05. *R^*2*^ = * 0.05, ^∗∗^*p* < 0.01, for word recognition; and *R^*2*^ = * 0.02, ^∗∗^*p* < 0.01, for working memory.*.

In summary, we found that word recognition and working memory related to and contributed to reading comprehension in the time constraint and non-time constraint reading conditions differently among Chinese college ELLs. The results indicated that varying reading time affected the relationship between word recognition, working memory, and reading comprehension. This is consistent with L1 findings and provided some empirical evidence for the first prediction of the Compensatory Encoding Model among FL readers.

### Discussion

The results of study one demonstrate that the correlations between word recognition, working memory, and reading comprehension in the time constraint and non-time constraint reading conditions differ from each other. The results were consistent with Walczyk’s (1995) study with L1 readers and extended the first prediction in the Compensatory Encoding Model that varying reading time affects relationship between word recognition efficiency, working memory capacity, and reading comprehension to FL reading. As indicated in the Compensatory Encoding Model, reading varying time may determine whether readers are able to orchestrate strategic processing successfully as a means to compensate for inefficient word recognition and working memory limitation. This seemed to be the case in FL reading with readers whose L1 and FL have different orthography.

No studies in FL reading have compared the time constraint and non-time constraint reading when they have examined the relationship between word recognition, working memory, and reading comprehension. However, research in FL reading on the relationship between word recognition and reading comprehension has produced inconsistent results: while some studies revealed that individual differences in word recognition did not lead to different levels of text comprehension in FL reading (e.g., Haynes and Carr, 1990; Shiotsu, 2009; Yamashita, 2013); other studies found that word recognition inefficiency adversely affected comprehension in FL reading (e.g., Nassaji and Geva, 1999; Nassaji, 2003a; Tsai, 2008). These inconclusive findings may be attributed to lack of control of reading time in these studies. The studies which found an inhibitory effect of inefficient word recognition might have readers complete reading tasks within restricted reading time in their design. For instance, Nassaji (2003a) and Nassaji and Geva (1999) reported a significant relationship between word recognition and reading comprehension for ELLs with advanced reading proficiency. The reading comprehension test used in the two studies is the Nelson–Denny Reading Test, a standardized L1 reading test for native English speakers. Thus, the test might be challenging for the ELLs, hence reading the texts might require additional time than that had been given to their participants.

On the other hand, those studies which reported no relationship between word recognition and reading comprehension might have given readers ample time to complete reading tasks. For example, Haynes and Carr (1990) found that individual differences in word recognition did not lead to different levels of text comprehension in FL reading among Chinese ELLs. In this study, the participants were asked to read a 500-word text and to record their reading time, which in fact, allowed students read at their own pace. Lack of control for time in reading might simulate the non-time constraint reading, which is essential for readers to utilize strategic repertoire as compensation for slow word recognition. Similarly, among Japanese ELLs, Yamashita (2013) also allowed readers to read in their own pace and recorded readers’ reading rates, and she found that word recognition was only significantly related to reading rate but not with reading comprehension.

Our results that varying time affects the relationship between word recognition efficiency and reading comprehension may also offer some explanations as to why some intervention studies on word recognition training failed to show any effect of increased word recognition on reading comprehension (e.g., Fukkink et al., 2005; Akamatsu, 2008). Even though the participants in the Fukkink et al. (2005) improved word recognition efficiency after intervention, their reading comprehension did not show any improvement. As explained by the researchers that “t[T]here was no time limit for completing the reading test” (Fukkink et al., 2005, p. 64), and we speculate this could be the reason for failure of showing the effect of increased word recognition efficiency on text comprehension. In the future, researchers may consider controlling reading time when testing comprehension in order to more accurately reflect the effect of word recognition training.

We found that even in the time constraint reading condition, word recognition efficiency and working memory could only make small contributions to reading comprehension. As reading is a highly complex cognitive activity and comprehension depends substantially on other skills in addition to word recognition and working memory, such as L1 reading ability, metacognitive knowledge, and FL proficiency (e.g., grammatical and vocabulary knowledge) (van Gelderen et al., 2003, 2004; Koda, 2005; van Gelderen et al., 2007; Grabe, 2009; Grabe and Stoller, 2011). Previous studies demonstrated that linguistic proficiency and metacognitive knowledge make much more substantial contribution than word recognition and sentence processing speed to FL reading (e.g., van Gelderen et al., 2003, 2004, 2007). Thus, it was reasonable to expect weak predictive power of word recognition and working memory.

In terms of the relationship between working memory and FL reading comprehension, our research found that students who have a larger working memory were more likely to achieve better comprehension in FL reading. The significant relationship in our research is in line with previous FL reading studies, such as Harrington and Sawyer’s (1992), and Walter’s (2004) studies, though the strength of the relationship in our research is much weaker than theirs (Harrington and Sawyer: *r* = 0.57; Walter: *r* = 0.79 for lower-intermediate group; *r* = 0.46 for higher-intermediate group). One possible reason for the difference could be the different instruments used for measuring working memory. Both Harrington and Sawyer’s and Walter’s used the RST to measure working memory, which largely depends on one’s reading ability, and the working memory tends to be confounded with reading comprehension ability (Seigneuric et al., 2000; Koda, 2005), hence may be responsible for much stronger association. Our research used the OST to measure working memory, which reduced the involvement of reading ability to its minimum. Therefore, the significant and small relationship between working memory and FL reading comprehension in our research represents the true relationship without being confounded. In summary, study one extended the first prediction in the Compensatory Encoding Model for L1 reading to FL reading. Whether FL readers are able to deploy compensatory use of reading strategies for word recognition inefficacy and working memory limitations will be investigated in study two.

## Study Two

### Material and Methods

#### Participants

The participants in study two were 30 second year undergraduates (14 males and 16 females) recruited from the same university as in study one. They were between 18 and 23 years old with an average of 20.57 years (*SD* = 1.10). Similarly, the average year of English learning for them was 7.5 years.

#### Materials

The materials used in study two were: the word recognition test, the working memory test, a reading passage for think-aloud, and the comprehension test. As the word recognition test and the working memory test were exactly the same as in study one, please see study one for the details.

##### The reading passage for think-aloud

We used the text – *Ideas about Beauty* – to collect think-aloud data. This text was T3 used in the non-time constraint reading condition in study one. The reason for using a text from the non-time constraint reading condition was that the think-aloud method gave the readers ample time to process a text, simulating a non-time constraint reading condition.

Before think-aloud, the participants received detailed training in Chinese on how to verbalize their thoughts while reading and practiced think-aloud with a short expository text until they felt confident enough to carry out the think-aloud. To reduce demands on participants’ verbal ability, they were free to articulate in either Chinese or English, or a combination of both (see Appendix 3 for the instructions). The think-aloud sessions were audio-recorded using a Lenovo audio-recorder.

##### The reading comprehension test and scoring

For the reading comprehension test, we used exactly the same 10 multiple choice questions (5 literal comprehension questions and 5 inferential comprehension questions) for *Ideas about Beauty* as in the study one. A correct answer for one question received one point.

#### Data Collection Procedure

Data collection for study two took place in the students’ free time in a quiet office. Each student first completed a think-aloud session followed by the reading test. They then completed the word recognition and working memory tests using a Dell laptop. The duration for data collection of study two ranged between 42 and 76 min.

#### Data Analysis

The data analysis in study two began with coding the think-aloud data. We first established a coding scheme on the basis of the two sources: Walczyk (2000) and Stevenson et al. (2007), in combination with the strategies from the data (see Appendix 4 for the coding scheme and examples). Reading strategies were categorized into: (1) language-oriented strategy, (2) content-oriented strategy, (3) re-reading above word-level, (4) pausing above word-level, and (5) meta-comment. Language-oriented strategies are directed toward “understanding the linguistic code of the text”; whereas content-oriented strategies are related to “building a mental model of the global conceptual content of the text” (Stevenson et al., 2007, p. 121). Re-reading above word-level refers to reprocessing part of the text which is more than a word. Pausing above word-level is defined as an interruption of decoding for 3 s or more of silence (Walczyk et al., 2004). Meta-comment is referred to as a reader’s evaluation and reflection on his/her reading processes.

Language-oriented strategies comprised of translating, paraphrasing, grammatical problem-solving, discourse problem-solving, word processing problem-solving, and lexical inferencing. Translating occurs when part of the text is translated from English to Chinese. Paraphrasing is a strategy used to rephrase meaning of one or parts of a sentence by replacing some words using synonyms, or reorganizing sentence structures either in English or Chinese. Grammatical problem-solving is used to disambiguate grammatical issues, while discourse problem-solving is defined as solving referential and/or cohesive devices problems. Word processing problem-solving involves possible strategies for solving inefficient word recognition, namely pausing and sounding out at word-level or below. Lexical inferencing strategies are used to make an informed guess of the meaning of an unknown word (Oxford and Scarcella, 1994; Kuhn and Stahl, 1998; Fraser, 1999; Paribakht and Wesche, 1999; Nassaji, 2003b; Bengeleil and Paribakht, 2004).

Content-oriented strategies consisted of summarizing, interpreting, predicting, as well as questioning. Summarizing refers to recapping the gist of more than one sentence. Interpreting means integrating textual information with one’s background knowledge to arrive at inferences of contents and purposes of part or all of a text. Predicting is defined as foreseeing the contents ahead, and questioning means raising questions concerning concepts conveyed in a text.

We used episodes as the unit of coding to code the frequency of reading strategy use with the assistance of the NVivo 9.2, which allowed audio files to be coded directly and the coded data were transformed into Excel for subsequent analysis. An episode was defined as a period when a reader “is unbrokenly occupied with the same component process” (Stevenson, 2005, p. 150). An episode ends at either the start of a different reading strategy or at a long pause of 10 s or more (Stevenson et al., 2007). The inter-coder reliability was calculated on randomly selected 6 think-aloud data, accounting for 20% of the total data. The Cohen’s kappa was 0.79 for language-oriented strategies, 0.86 for content-oriented strategies, 0.88 for re-reading above word-level, 0.77 for pausing above word-level, and 0.79 for meta-comment. The frequency of reading strategy use was then correlated with word recognition, working memory, and reading comprehension to answer the research question of study two.

### Results

Altogether we identified a total of 2,339 strategies with an addition of seven unintelligible verbalizations, which were excluded from the analyses. The descriptive statistics of the five main types of strategy are displayed in **Table 5**, which shows that on average, our participants used 78 strategies to complete the reading task but the actual number of strategies used by the participants varied considerably as shown by a large *SD* of 30.10.

**Table 5:** Frequency of the five types of reading strategy.

Types of reading strategy	Minimum	Maximum	*M*	*SD*
Language-oriented strategy	3	52	25.20	10.98
Content-oriented strategy	2	27	12.03	6.52
Re-reading above word-level	2	30	12.90	6.79
Pausing above word-level	0	15	3.37	3.94
Meta-comment	0	18	5.37	5.52

A one-way repeated ANOVA and Bonferroni *post hoc* analysis were employed to examine if there were significant differences of strategy use. The ANOVA showed that the participants differed significantly in terms of frequency of strategy use, *F*(4,29) = 66.21, *p* < 0.01, $\eta_{p}^{2}$ = 70. Bonferroni pair-wise comparison indicated that among the five main categories of reading strategy, the participants employed language-oriented strategies most frequently (*M* = 25.20, *SD* = 10.98). They used content-oriented strategies (*M* = 12.03, *SD* = 10.98) and re-reading above word-level (*M* = 12.90, *SD* = 6.79) less than half as frequently as language-oriented strategies. But there was no significant difference between content-oriented strategies and re-reading above word-level. The least frequently used reading strategies were pausing above word-level (*M* = 3.37, *SD* = 3.94) and meta-comment (*M* = 5.37, *SD* = 5.52), which did not differ from each other.

**Table 6** presents the results of correlation analyses between word recognition, working memory, frequency of five types of reading strategy use, and reading comprehension. The relationship between word recognition and different kinds of reading strategies indicated that word recognition was significantly and positively correlated with language-oriented strategy use (*r* = 0.71, *p* < 0.01), re-reading above word-level (*r* = 0.40, *p* = 0.03), and pausing above word-level (*r* = 0.53, *p* < 0.01); whereas the association between word recognition and content-oriented strategy use (*r* = 0.33, *p* = 0.08) and meta-comment (*r* = 0.33, *p* = 0.24) were non-significant. This means that the students who had longer time to recognize English words (i.e., slower in word recognition) were associated with using more language-oriented strategy, re-reading above word-level, and pausing above word-level.

**Table 6:** Correlation analyses between reading strategy use, word recognition, working memory, and reading comprehension.

Reading strategy use	Word recognition	Working memory	Comprehension
Language-oriented strategy	0.71^∗∗^	-0.50^∗∗^	0.77^∗∗^
Content-oriented strategy	0.33	-0.33	0.20
Re-reading above word-level	0.40^∗∗^	-0.57^∗∗^	0.54^∗∗^
Pausing above word-level	0.53^∗∗^	-0.35	0.43^∗∗^
Meta-comment	0.33	-0.24	0.33

*^∗∗^*p* < 0.01 (two-tailed)*.

Resembling the results between word recognition and types of reading strategy use, we found that working memory did not significantly relate to content-oriented strategy use (*r* = -0.33, *p* = 0.07) and meta-comment (*r* = -0.24, *p* = 0.21), but it had significant and moderate association with language-oriented strategy use (*r* = -0.50, *p* < 0.01) and re-reading above word-level (*r* = -0.57, *p* < 0.01), suggesting that readers with a smaller working memory tended to use language-oriented strategy and paused more frequently. The correlation between working memory and pausing above word-level was also not significant (*r* = -0.35, *p* = 0.06).

Furthermore, we found that reading comprehension was significantly and positively correlated with language-oriented strategy use (*r* = 0.77, *p* < 0.01), re-reading above word-level (*r* = 0.54, *p* < 0.01), and pausing above word-level (*r* = 0.43, *p* = 0.02). However, neither the association between reading comprehension and content-oriented strategy use (*r* = 0.20, *p* = 0.30) nor the association between reading comprehension and meta-comment (*r* = 0.33, *p* = 0.08) was significant.

### Discussion

The interrelationship between word recognition, working memory, strategy use, and reading comprehension demonstrated that when FL readers had sufficient time to process a reading task at hand, the more frequently they adopted language-oriented strategy, re-reading above word-level, and pausing above word-level, the better comprehension they achieved, and the reading comprehension was not affected by the performance of word recognition and working memory capacity. On the other hand, the content-oriented strategy use and meta-comment did not function as compensation for word recognition inefficiency and limited working memory. As described in the Compensatory Encoding Model, the kinds of strategies which are compensatory are those ones which mostly focus on solving language problems, re-reading, and pausing (Walczyk, 2000), just as what we found in our study, rather than strategies which focus on helping with understanding the global comprehension (content-oriented) and focus on the evaluation of their reading processes and behaviors (meta-comment).

Study two shows that the participants had a wide repertoire of reading strategies to draw on during FL reading, though the frequency of different types of reading strategies varied. Among five main categories of strategies classified, three of them, namely language-oriented strategy, re-reading above word-level, and pausing above word-level, were similar to the strategies with compensatory nature described in the Compensatory Encoding Model (Walczyk, 2000).

Regarding the relationship between word recognition, working memory, and use of reading strategies, our results of study two lent support to the second prediction of the Compensatory Encoding Model that when readers read without time restriction, word recognition efficiency and working memory tend to adversely relate to frequency of reading strategy use among Chinese college ELLs. This means that FL readers also displayed similar manner of using strategies to L1 readers to compensate for inefficiency in word recognition and limited working memory capacity so that their comprehension was not affected when they read in non-time constraint reading. However, we found that word recognition and working memory did not relate to all kinds of reading strategies, and content-oriented strategies and meta-comments seemed not to be compensatory. This finding was in fact in line with the Compensatory Encoding Model, in which the compensatory mechanisms are predominantly used to solve language problems rather than to direct toward understanding global conceptual comprehension (Walczyk and Taylor, 1996; Walczyk, 2000; Walczyk et al., 2001, 2007).

A possible reason why re-reading and pausing appeared to be effective compensatory strategies in FL reading comprehension could be explained by Kintsch’s (1998) construction-integration perspective of comprehension. When readers’ word recognition is not efficient enough, the initial derivation of propositional meanings from these processes tends to take up many working memory resources to construct a text-base of comprehension (the construction phase). By the end of the construction phase, readers’ working memory may be exhausted, and this in turn leaves them with insufficient working memory resources to construct a situation-base of comprehension (the integration phase). In order for readers to have enough working memory in the integration phase, they may need to pause and re-read often in order to enable integration to take place. The reason that language-oriented strategies rather than content-oriented strategies were compensatory in FL text comprehension could be that the global conceptual understanding in reading largely depends on a reasonable understanding of the local meaning construction of the text (Kintsch, 1994, 1998). Thus, without achieving a good comprehension of phrases and sentences at the local level, it would be hard for FL learners to apply strategies toward building a coherent representation of the text.

The results of study two corroborated with studies in L1 reading with both children and adults (e.g., Walczyk and Taylor, 1996; Walczyk et al., 2001, 2004, 2007). The findings were also consisted with those in Stevenson’s (2005) study. Stevenson also reported that among Dutch middle school ELLs, speed of recognizing English words was significantly related to language-oriented strategies but not to content-oriented strategies, and levels of reading comprehension. The similar results between the two studies suggest that language distance between L1 and FL in terms of orthography does not affect readers’ successful use of reading strategies – a higher-order process in reading – to compensate for in efficient word recognition – a lower order process in reading. This appears to support Taylor and Taylor’s (1995) assertion that processing strategies at the word level tend to be affected by differing orthographies across languages, but reading strategies at the higher-order comprehension processes tend to remain similar.

### Conclusion

To sum up, our research provided empirical evidence for the support of the Compensatory Encoding Model among Chinese ELLs. Despite that Chinese ELLs’ English word recognition skills tend to be adversely affected by their holistic approach used in Chinese word recognition (Muljani et al., 1998; Akamatsu, 2003, 2005; Wang and Koda, 2005; Hamada and Koda, 2008, 2010), their compensatory reading strategy use (i.e., higher order skills) resembles that of native English speakers (e.g., Walczyk and Taylor, 1996; Walczyk et al., 2001, 2004) and ELLs whose L1 is also an alphabetic language (Stevenson, 2005). Our research indicates that rather than supporting the popular beliefs that word recognition efficiency is important in FL reading comprehension (Koda, 1996; Segalowitz, 2000), we found that higher-order metacognitive strategy use is more important and predictive in FL reading comprehension, especially when readers are given ample time to process a reading task. As we found that language-oriented strategies are useful compensatory strategies, FL reading educators may wish to design training programs to teach students to use these strategies in order to facilitate text comprehension. The findings that contributions made by word recognition and working memory to FL reading comprehension are affected by reading varying time imply that time-restricted FL reading comprehension assessments may limit students’ opportunities to apply some strategies to compensate for word processing inefficiency and cognitive resource limitation. Therefore, FL reading assessments may need to include multiple ways to measure FL readers’ reading comprehension ability.

## Ethics Statement

This study was carried out in accordance with the recommendations of ‘National Statement on Ethical Conduct in Human Research, the Human Research Ethics Committee of the University of Sydney’ with written informed consent from all subjects. All subjects gave written informed consent in accordance with the Declaration of Helsinki. The protocol was approved by ‘the Human Research Ethics Committee of the University of Sydney.’

## Author Contributions

The author confirms that she contributed to this paper by: Contributing substantially to the conception of the work; and the acquisition, analysis, and interpretation of the data; Drafting the work and revising it critically for important intellectual content; Approving the final version of the paper to be published; Agreeing to be accountable for all aspects of the work in ensuring that questions related to the accuracy or integrity of any part of the work are appropriately investigated and resolved.

## Conflict of Interest Statement

The author declares that the research was conducted in the absence of any commercial or financial relationships that could be construed as a potential conflict of interest.

## Footnotes

1 www.thesaurus.com
