# Corrigendum: Applicability of the Compensatory Encoding Model in Foreign Language Reading: An Investigation With Chinese College English Language Learners

**DOI:** 10.3389/fpsyg.2018.00919

**Published:** 2018-06-19

**Authors:** Feifei Han

**Affiliations:** Centre for Research on Learning and Innovation, Sydney School of Education and Social Work, The University of Sydney, Sydney, NSW, Australia

**Keywords:** compensatory encoding model, word recognition, working memory, reading comprehension, foreign language reading, Chinese college English language learners

The original Appendix 3, which contained the four reading texts, was removed due to copyright concerns.

The enumeration of the appendices has been updated accordingly. The author apologizes for this error and states that this does not change the scientific conclusions of the article in any way. The original article has been updated.

## Conflict of interest statement

The author declares that the research was conducted in the absence of any commercial or financial relationships that could be construed as a potential conflict of interest.

