# Supplementary material for: Applicability of the Compensatory Encoding Model in Foreign Language Reading: An Investigation with Chinese College English Language Learners
Source: Front Psychol. 2017 May 4;8:681. doi: 10.3389/fpsyg.2017.00681 (PMC5415605; doi:10.3389/fpsyg.2017.00681)
Supplement: Supplementary file 1 [file Data_Sheet_1.docx]

**Appendix 1 Sample Word Recognition Test Items**

| **Item no.** | **Synonym word pairs** | | | **Part of speech** |
| --- | --- | --- | --- | --- |
| 1 | near | | close | adj. |
| 2 | begin | | start | v. |
| 3 | immediately | | soon | adv. |
| 4 | street | | road | n. |
| **Item no.** | | **Antonyms word pairs** | | **Part of speech** |
| 1 | after | | before | adv. |
| 2 | good | | bad | adj. |
| 3 | top | | bottom | n. |
| 4 | forget | | remember | v. |

**Appendix 2 Sample Working Memory Test Items**

| **Item no.** | **Words for recall** | **Equations** | **Answer** |
| --- | --- | --- | --- |
| 1 | start | (9 / 1) + 4 = 14 correct? | No |
| 2 | room | (6 / 2) -2 = 1 correct? | Yes |
| 1 | read | (9 x 1) + 9 = 16 correct? | No |
| 2 | cost | (8 x 1) + 5 = 13 correct? | Yes |
| 3 | care | (6 x 2) -3 = 10 correct? | No |
| 4 | foot | (8 / 4) -1 = 1 correct? | Yes |
| 1 | girl | (3 / 3) + 5 = 7 correct? | No |
| 2 | bring | (10 / 1) + 2 = 12 correct? | Yes |
| 3 | love | (10 / 1) -1 = 9 correct? | Yes |
| 1 | side | (6 x 1) -4 = 1 correct? | No |
| 2 | hear | (10 / 2) -3 = 2 correct? | Yes |
| 3 | speak | (5 x 2) -5 = 4 correct? | No |
| 4 | mind | (2 x 1) -3 = 6 correct? | No |
| 5 | month | (7 / 1) -2 = 5 correct? | Yes |

**Appendix 3 Instructions of Think-aloud**

1. Today you are going to read one English text. Try to understand the text as well as possible. While you are reading the text, I’d like you to say out aloud everything that comes to your mind. The reason I want you to do this is that I am interested in finding out what students do and what they think about in order to understand a text.

2. You’re probably not used to saying out aloud everything while you read. It goes like this:

You read the text aloud. The important thing is to read the text the way that it is easiest for you to understand it. How well the text is read aloud is NOT important. It doesn’t matter if you don’t pronounce the words correctly or if you don’t read quickly.

While you read, keep on saying everything that you’re doing and everything that you’re thinking about. It doesn’t matter at all if you stop half way through a sentence to say something. You can say things about the text, like:

“I don’t know what the writer’s message is. I think he’s trying to tell us…”

“What else is the text going to be about? I’ll read on…”

“I don’t agree with what the writer says.”

You can also say things about the words and sentences in the text, like:

“I don’t understand this sentence. I’ll read it again.”

“What does that word mean? I think it means…”

“Let me think. Yes, I understand what that word means”.

3. It doesn’t matter in which language you say: You can use Chinese, English or both as long as you feel comfortable.

4. It’s okay if you want to re-read some of the text or to read ahead in the text. You can do anything that helps you understand the text well.

5. If you stop saying out everything aloud, I’ll ask: “What are you thinking about?” Apart from this, I won’t say anything. I can’t answer questions and I can’t say anything to help you understand the text. I also can’t tell you if the things you say about the text are right or not. NOTE: There is actually no right or wrong, so just say whatever you’re thinking about.

6. Before you start, I will give you a short paragraph to practise saying everything aloud while reading for comprehension. This practice session won’t be audio recorded.

7. After this practice, you’ll have the opportunities to ask questions for clarification.

8. Please speak loudly enough so that your voice can be recorded.

**Appendix 4 Coding scheme and examples**

| **Main categories** | **Categories** | **Sub-categories** |
| --- | --- | --- |
| 1 language-oriented strategies | 1.1 translating |  |
|  | 1.2 paraphrasing |  |
|  | 1.3 grammatical problem-solving |  |
|  | 1.4 discourse problem-solving |  |
|  | 1.5 word processing problem-solving | 1.5.1 pausing |
|  |  | 1.5.2 sounding out |
|  | 1.6 lexical inferencing |  |
| 2 content-oriented strategies | 2.1 summarizing |  |
|  | 2.2 interpreting |  |
|  | 2.3 predicting |  |
|  | 2.4 questioning |  |
| 3 re-reading above word-level |  |  |
| 4 pausing above word-level |  |  |
| 5 meta-comment |  |  |

**Examples of Reading Strategies**

Examples of the categories and sub-categories of reading strategies in the coding scheme are shown below. *Italics* *words* in quotation marks mean the words from the reading texts, **bold** **words** in quotation marks mean Chinese.

**1. language-oriented strategies**

**1.1 translating**

e.g., “*Most people would agree*... d**aduoshu ren dou tongyi**” in Chinese (majority people all agree).

**1.2 paraphrasing**

e.g., “*In fact, Dr. Singh found that any woman whose waist is 70% as wide as her hips is judged as beautiful by most men no matter how big the woman in overall.* This sentence means...Dr. Singh discovered that even if a woman is fat, as long as her waist is around 70% of her hips, men will like her.”

**1.3 grammatical problem-solving**

e.g., “*Evolution would favour men who have inherited genes from their fathers,* in this sentence *men who have inherited genes from their fathers*, this bit modifies men.”

**1.4 discourse problem-solving**

e.g., “*However, researchers do not agree on whether the factors which influence*…the word *however*, so the author is saying something different from the previous sentence.” (cohesive device)

“*They seemed not to prefer women with ‘hourglass’ shapes*…*they* here refers to men in isolated communities, not men in a general sense as mentioned before.” (referential problems)

**1.5 word processing problem-solving**

**1.5.1 pausing**

e.g., “Hour...hour...hour...glass”.

**1.5.2 Sounding out**

e.g., “This word, t-a...no tr-a-m-i-d”.

**1.6 lexical inferencing**

e.g., “a psychologist, I think this means some kinds of people who are experts in a field, like biologists; I’ve learnt the meaning of this ending -ist.”

**2. content-oriented strategies**

**2.1 summarizing**

e.g., “The first paragraph opens up what the author’s focus of the passage, something is related to beauty.”

**2.2 interpreting**

e.g., “The psychologist did an experiment. Yeah, I think this scientist wanted to use this experiment to support or prove his theory. You know like everywhere, if you have a theory, you’re going to do something, like...to find evidence and to show people that your theory is correct.”

**2.3 predicting**

e.g., “*Devendra Singh…conducted an experiment in 1993*…I guess the author uses this experiment to illustrate his argument in the first paragraph. I don’t know, let me continue to read.”

**2.4 questioning**

e.g., “prefer heavier women, eh, heavier? People like thin women nowadays, heavier? Ah, yes, these people don’t like thin women.”

**3. re-reading above word-level**

e.g., “*beauty is the eye of the beholder...beauty is the eye of the beholder*”.

**4. pausing above word-level**

e.g., “*Women who develop an hourglass shape...*” (silent).

**5. meta-comment**

e.g., “I don’t understand the meaning of this sentence.”
